# Supplementary material for: A set of multi-entry identification keys to African frugivorous flies (Diptera, Tephritidae)
Source: Zookeys. 2014 Jul 24;(428):97–108. doi: 10.3897/zookeys.428.7366 (PMC4143993; doi:10.3897/zookeys.428.7366)
Supplement: Supplementary material 4 — Key to Capparimyia [file zookeys-428-097-s004.zip › SF4_ZooKeys_key to Capparimyia/lucid_player/help/help/the_applet_player_and_application_player.htm]

The Applet Player and Application Player


# The Applet Player and Application Player

Lucid3 keys may be deployed using an Application or an Applet Player.
The Application Player is a standalone version installed to a desktop
computer. The Applet Player is a smaller version that can be embedded
in web pages; when the web page is visited, the applet is downloaded from
the web server (as part of the web page within your browser) then loads
the Lucid3 key.

Although the Applet and Application Players work in very similar ways,
there are minor differences. In this Help file, wherever operations in
the Application and Applet Player differ, the text is annotated; if the
type of Player is not specified, the description applies to both Applet
and Application.

The Lucid3 application player

The Lucid3 applet player
